# Supplementary material for: Asymmetric tapered multistage solar still with optimized mass transfer equilibrium for ultrahigh water production
Source: Nat Commun. 2025 Nov 12;16:9950. doi: 10.1038/s41467-025-64897-8 (PMC12612217; doi:10.1038/s41467-025-64897-8)
Supplement: Supplementary file 1 — Supplementary Information [file 41467_2025_64897_MOESM1_ESM.pdf]

Supplementary Information

Asymmetric tapered multistage solar still with optimized mass transfer equilibrium for ultrahigh water production

Wen He[1]†, Jiacheng Wang[2]†, Baiyi Chen[3]†, Yiyao Li[4], Zhongyi Fang[1], Xuan Zhou[1], Lei Zhou[4], Meng Li[2], Xu Hou[1,4,5] *

[1] State Key Laboratory of Physical Chemistry of Solid Surfaces, College of Chemistry and Chemical Engineering, Xiamen University, Xiamen 361005, China.

[2] Key laboratory of Low-grade Energy Utilization Technologies & Systems, Ministry of Education, CQU-NUS Renewable Energy Materials & Devices Joint Laboratory, School of Energy & Power Engineering, Chongqing University, Chongqing 400044, China.

[3] Marine Engineering College, Fujian Provincial Key Laboratory of Advanced Marine Functional Materials, Xiamen Key Laboratory of Marine Corrosion and Smart Protective Materials, Jimei University, Xiamen 361021, China.

[4] College of Physical Science and Technology, Xiamen University, Xiamen 361005, China.

[5] Engineering Research Center of Electrochemical Technologies of Ministry of Education, Xiamen University, Xiamen 361005, China.

*Corresponding author. Email: houx@xmu.edu.cn
†Equally contributed to this work.

Contents:

1. Supplementary Notes

Supplementary Note 1: Calculation of production rate and conversion efficiency
Supplementary Note 2: Salt rejection rate
Supplementary Note 3: Liquid seal component
Supplementary Note 4: The calculation of heat loss of single-stage device
Supplementary Note 5: The heat transfer process of multistage device

2. Supplementary Methods

Supplementary Methods 1: COMSOL simulation

Supplementary Methods 1: Ray tracing Simulation

3. Supplementary Figures

Supplementary Fig. 1: The light absorption and photothermal performance
Supplementary Fig. 2: The microscopic images of the evaporation and condensation layer
Supplementary Fig. 3: The condensation behavior of vapor on different surfaces
Supplementary Fig. 4: The diagram of cone angle
Supplementary Fig. 5: The geometric parameters single-stage device with different conical apex angle
Supplementary Fig. 6: The vapor production in open system (J_O) and closed chamber (J_C)
Supplementary Fig. 7: The parasitic heat transfer of single-stage device with different mass transfer gap
Supplementary Fig. 8: Two-dimensional geometrical model of the ATMSMD system
Supplementary Fig. 9: Vapor distribution in the mass transfer chamber at different time intervals
Supplementary Fig. 10: The diagram and photograph of the mechanism of liquid seal
Supplementary Fig. 11: The vapor and water production of ten-stage asymmetric tapered solar still with a uniformed D of 8 mm
Supplementary Fig. 12: The performance of optimized eight-stage asymmetric tapered solar still with a uniformed mass transfer gap (D) of 8 mm
Supplementary Fig. 13: The input energy and heat loss of each stages
Supplementary Fig. 14: The water production of each stage in eight-stage device with different D
Supplementary Fig. 15: The comparison of 8-stage asymmetric tapered solar still with a uniformed D of 8 mm and with a gradually decreasing D
Supplementary Fig. 16: The diagram of the setup under 1-sun illumination with different α.
Supplementary Fig. 17: The weather parameters of the three days with different climate conditions
Supplementary Fig. 18: The water production of the three days with different climate conditions
Supplementary Fig. 19: The photograph of scaled-up eight-stage device.
Supplementary Fig. 20: The light intensity and water production at the noon of three sunny days.
Supplementary Fig. 21: The collected brine in the low-level side during the dark.

4. Supplementary Tables

Supplementary Table 1: The parameters of single-stage device with different D
Supplementary Table 2: The parameters of multistage device
Supplementary Table 3: The data of state-of-the-art multistage solar membrane distillation devices used for comparison
5. Supplementary Reference

Supplementary Notes

1. Calculation of production rate and conversion efficiency

The energy efficiency addressed in this work includes the solar-to-vapor, solar-to-water, and vapor-to-water efficiency. The vapor production rate is calculated by

$$J = \frac{m_v}{At} \quad (1)$$

where, m_v is mass of the vapor, A is the projected area, t is the time required to evaporate water. The vapor production rate is calculated by

$$J_{prod} = \frac{m_w}{At} \quad (2)$$

where, m_w is mass of the condensed water.
The vapor-to-water conversion efficiency η_c is calculated by

$$\eta_c = \frac{m_w}{m_v} \quad (3)$$

2. Salt rejection rate.

The salt rejection rate experiments were performed at natural seawater. The initial concentrations (C_0) and the concentration after desalination (C_1) of Na^+, K^+, Ca^{2+}, Mg^{2+} and B^{3+}.

The salt rejection rate was calculated using equation (4):

$$R = \left(1 - \frac{C_1}{C_0}\right) \times 100\% \quad (4)$$

3. Liquid seal component

We polished the originally hydrophobic-modified inner surface of the tapered bottom to create a hydrophilic region approximately 4 ~ 5 mm in height, allowing liquid to form a concave meniscus at the bottom. The Laplace pressure (Δp) of the concave meniscus balances with the supporting force ($\mathbf{F_s}$) from the inclined surface and the gravitational force ($\mathbf{G}$), stabilizing the droplets at the pore opening of the tapered top. As condensation increases, more water accumulates at the bottom of the taper, causing gravity to exceed the combined Laplace pressure Δp and $\mathbf{F_s}$, leading to downward liquid flow. When the liquid volume decreases, the system returns to equilibrium, and the liquid stabilizes again on the tapered surface.

4. The heat transfer process of single-stage device

The conduction (q_{cond}), convection (q_{conv}), radiation (q_{rad}) and sidewall heat loss (q_{side}) of the single-stage device using the following equations:

$$q_{cond} = k_{vapor} \cdot A_{gap} \cdot \frac{T_e - T_c}{D} \quad (5)$$

$$q_{conv} = h_{conv} \cdot A_e \cdot (T_e - T_a) \quad (6)$$

$$q_{rad} = \epsilon_{eff} \cdot \sigma \cdot A_e \cdot (T_e^4 - T_a^4) \quad (7)$$

$$\epsilon_{eff} = \frac{1}{\frac{1}{\epsilon_e} + \frac{1}{\epsilon_a} - 1} \quad (8)$$

where k_{vapor} is the thermal conductivity of water vapor (about 45°C, 0.0195 W·m^{-1}·K^{-1}), A_e is the area of evaporation surface, A_{gap} is the average cross-sectional area of mass transfer gap, T_e is the temperature of evaporation surface, T_c is the temperature of condensation surface, T_a is the ambient temperature, D is the size of mass transfer gap, h_{conv} is the convective heat transfer coefficient (5 W·m^{-1}·K^{-1}), ϵ_e and ϵ_c are the emissivity of the evaporation and condensation surface.

$$q_{side} = \frac{T_{avg} - T_a}{R_{side}} \cdot A_{side} \quad (9)$$

$$T_{avg} = \frac{T_e + T_c}{2} \quad (10)$$

and the total thermal resistance R_{side} is given by:

$$R_{side} = \frac{t}{\kappa_{insul}} + \frac{1}{h_c} \quad (11)$$

where A_{side} is the area of sidewall, t is the insulation layer thickness, κ_{insul} is the thermal conductivity of the insulation material (the PS foam used in this work: 0. 044 W·m^{-1}·K^{-1}), h_c is the external convection heat transfer coefficient (assumed to be 10 W·m^{-1}·K^{-1}).

5. The heat transfer process of multistage device

Given the structural complexity and limited thermal conduction between isolated stages, we employed a simplified steady-state model in which the dominant thermal dissipation mechanism is assumed to be sidewall heat loss from each stage to the ambient environment. In this model, we neglect inter-stage conductive heat loss and radiation heat loss, as the primary energy input to each stage is the latent heat released by condensation in the preceding stage. The majority of thermal losses in each stage arise from convective and radiative heat exchange through the sidewall surfaces, which are in contact with ambient air. Therefore, we approximate the total thermal loss in stage i as[1]:

$$q_{loss,i} \approx q_{side,i} = \frac{T_{avg,i} - T_a}{R_{side}} \cdot A_{side,i} \tag{12}$$

$$T_{avg,i} = \frac{T_{e,i} + T_{c,i}}{2} \tag{13}$$

and the total thermal resistance R_{side} is given by:

$$R_{side} = \frac{t}{\kappa_{insul}} + \frac{1}{h_c} \tag{14}$$

Where $A_{side,i}$ is the area of sidewall, t is the insulation layer thickness, κ_{insul} is the thermal conductivity of the insulation material (the PS foam used in this work: 0. 044 $W \cdot m^{-1} \cdot K^{-1}$), h_c is the external convection heat transfer coefficient (assumed to be 10 $W \cdot m^{-1} \cdot K^{-1}$).

This simplified model reveals that as the stage number increases, the average temperature $T_{avg,i}$ drops, leading to reduced absolute heat loss per stage. However, the fraction of the thermal input lost to the environment increases in later stages due to the diminishing energy available for evaporation. This behavior is consistent with the experimentally observed decrease in vapor-to-water conversion efficiency in later stages (Fig. 3h), and it ultimately defines the practical limit of usable stage numbers in passive multistage solar stills.

Overall, this thermal loss analysis confirms that sidewall dissipation is the dominant heat loss pathway in our system and highlights the importance of thermal confinement and stage-wise optimization in achieving high-efficiency water production.

Supplementary Methods

1. COMSOL simulation

The vapor concentration field in the ATMSMD system are investigated by creating a transient model using the commercial software COMSOL Multiphysics®. The vapor flow in the ATMSMD system is controlled by the turbulence equation, which is as follows：

$$\rho(\mathbf{u}\cdot\nabla)\mathbf{u} = \nabla\cdot[-p\cdot I + (\mu_T+\mu)(\nabla u + (\nabla u)^T)] + \mathbf{F} \quad (15)$$

$$\rho V\cdot\mathbf{u} = 0 \quad (16)$$

Where, ρ is fluid density; $\mathbf{u}$ is the velocity field in the fluid; p is the fluid pressure; I is turbulence intensity; μ_T is the turbulent viscosity coefficient; μ is the dynamic viscosity; T is the fluid temperature; $\mathbf{F}$ is an external force acting on a fluid. The turbulent viscosity coefficient (μ_T) is calculated by the following equation：

$$\mu_T = \rho C_\mu \frac{k^2}{\sigma_\varepsilon} \quad (17)$$

Where, C_μ is the model coefficient; k is turbulent kinetic energy; σ_ε is the Prandtl (Pr) number corresponding to the turbulent dissipation ε.

The fluid and solid heat transfer field is used to describe the phase transition and temperature change of water vapor in the unit due to temperature change. The governing equation is as follows:

$$\rho C_p\frac{\partial T}{\partial t} + \rho C_p\mathbf{u}\cdot\nabla T + \nabla\cdot q = 0 \quad (18)$$

$$q = -k\nabla T \quad (19)$$

Where, ρ is the density; C_p is the specific heat capacity; T is the temperature; k is thermal conductivity; q is the heat flux. The two fields are coupled by non-isothermal flow, that is, the flow of water vapor is coupled with the temperature change, that is, the phase change.

Condensing unit energy conservation equation is as follows:

$$\frac{\partial(\rho h)}{\partial t} + \nabla(\rho\mathbf{u}h) - \nabla(\frac{\lambda}{C_p}\nabla T) - S_T = 0 \quad (20)$$

Where, h is the specific enthalpy of the fluid; λ is the thermal conductivity; C_p is the specific heat capacity; S_T is the energy source term.

Boundary conditions:

1) The inlet boundary conditions of the steam flow zone are given by setting the inlet velocity:

$$\mathbf{u} = \mathbf{u_{in}} = 0.002$$

2) The outlet boundary conditions are given by setting the pressure outlet:

$$p = p_{out} = 0$$

3) The temperature outside the condensing tube is T_{ext} = 278.15K, and the type of heat transfer outside the tube is forced convection heat transfer around the cylinder.

Geometry:

The ATMSMD system can be simplified as a two-dimensional model (Supplementary Fig. 8a), which comprises three primary components: an evaporation layer, an air gap, and a condenser layer. The thickness of the evaporation layer is set to 2 mm, the air gap is varied to 2 mm, 5 mm, and 8

mm in different cases, and the condenser layer has a thickness of 1 mm.

2. Ray tracing Simulation

(1) Geometric Model Construction

The three-dimensional model of the conical evaporator was constructed within TracePro 7.3 using its native solid modeling tools. The key geometric parameters are defined as follows:

Cone Geometry: The evaporator is modeled as a right circular cone. The slant height (L) is 50 mm, and the base diameter (d) is 50 mm. The apex angle is 60°, the height (H) of the cone is therefore 43.3 mm.

Material Definition: The cone body is assigned the optical properties of CNTs-coated Al foil. The inner surface of the cone is defined as the optical absorption surface.

Simulation Domain: The cone is positioned within a large, hollow rectangular air volume. The dimensions of this air volume (5×d × 5×d × 5×H) are significantly larger than the cone itself to ensure all stray rays are fully traced without unintended boundary interactions.

(2) Definition of Optical Properties and Surface Characteristics

The accuracy of the ray-tracing simulation critically depends on the precise definition of the optical properties for all surfaces involved.

Absorber Surface: The inner surface of the cone was defined as an ideal Lambertian (diffuse) reflector. This assumption is based on the typical micro/nano-structured nature of photothermal evaporation surfaces, which promotes diffuse scattering and multiple reflections to enhance light trapping. The surface reflectance (R), absorptance (A), and transmittance (T) were set as R = 0.04, A = 0.96, T = 0.00, ensuring R + A + T = 1.

(3) Light Source and Ray Tracing Setup

Source Type: A Collimated Beam source was used to simulate incident sunlight. The beam profile was defined as a circular disk with a diameter sufficiently larger than the base diameter of the cone to ensure complete illumination of the aperture under all incidence angles.

Incidence Angles: Three primary incidence conditions were investigated:

Normal Incidence (0°): The beam direction vector is parallel to the central axis of the cone.

Oblique Incidence (30° and 60°): The direction of the collimated beam was rotated by 30° and 60° relative to the central axis.

Ray Count: To ensure statistical convergence and result stability, a total of N = 1,00,000 rays were launched for each simulation case. A convergence test was performed by monitoring the total absorbed flux as a function of the number of rays; the result variation was found to be less than 0.5% beyond N = 50,000 rays, confirming that N = 1,00,000 was sufficient.

Wavelength: The simulation was performed using a monochromatic source at a wavelength of λ = 500 nm, corresponding to the approximate peak intensity of the standard AM 1.5G solar spectrum.

(4) Simulation Execution and Analysis

Ray Tracing Algorithm: TracePro employs a Monte Carlo method to trace rays through the optical system. The software calculates the path of each ray, accounting for specular and diffuse reflections, transmissions, and absorptions based on the defined surface properties and the probability functions derived from them.

Critical Output: The primary metric of interest was the total power absorbed by the inner surface of the conical evaporator. This value is directly reported by TracePro's built-in flux calculation tool.

Supplementary Figures

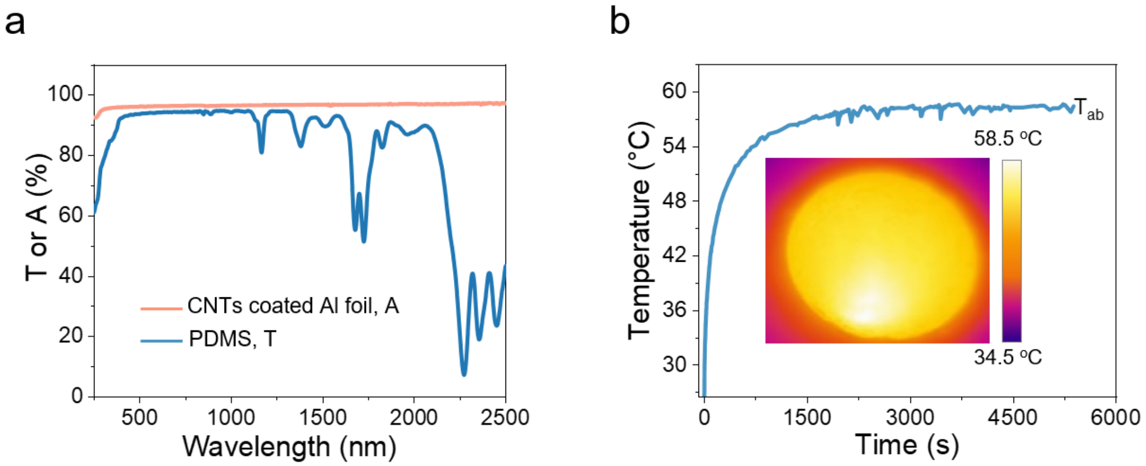

Supplementary Fig. 1 | The light absorption and photothermal performance. a, The transmittance of convection blocker and the absorbance of photothermal layer. **b,** The temperature change and infrared image of the photothermal layer. The inset is the infrared thermal image of the absorber under 1 kW·m^{-2}.

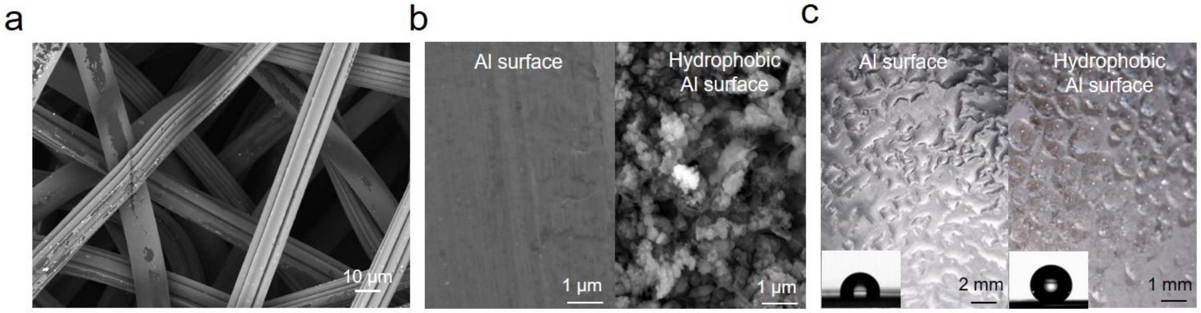

Supplementary Fig. 2 | The microscopic images of the evaporation and condensation layer. **a,** The SEM image of evaporation layer. **b,** The SEM images of Al and hydrophobic Al surface. **c,** The condensation behavior of Al surface and hydrophobic Al surface. The insets are the contact angle photographs of the two surfaces.

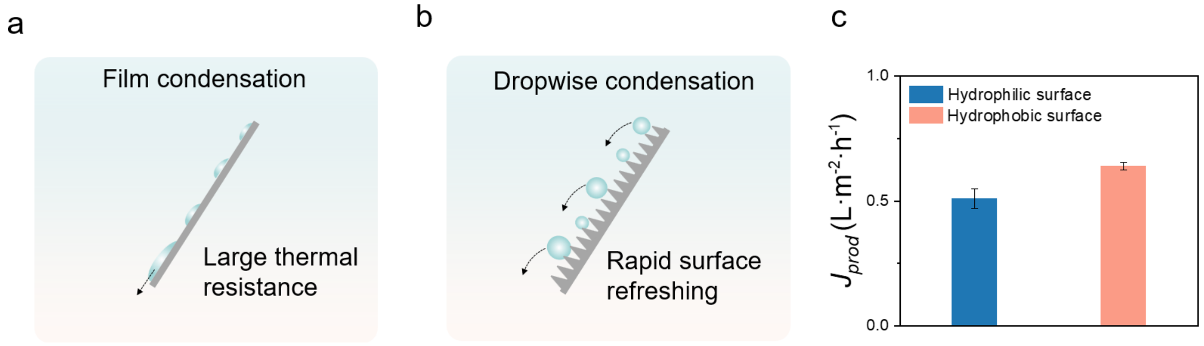

Supplementary Fig. 3 | The condensation behavior of vapor on different surfaces. a, The film condensation behavior. Condensed water tends to form a continuous film on the surface and slides down under gravity. **b,** The dropwise condensation behavior. Condensed water tends to form discrete droplets on the surface, which subsequently bounce off. **c,** The water production of the devices using the hydrophobically modified aluminum foil and aluminum foil as condensation surface. Error bars in c represent the s.d. (n = 3) and data are presented as mean values ± s.d.

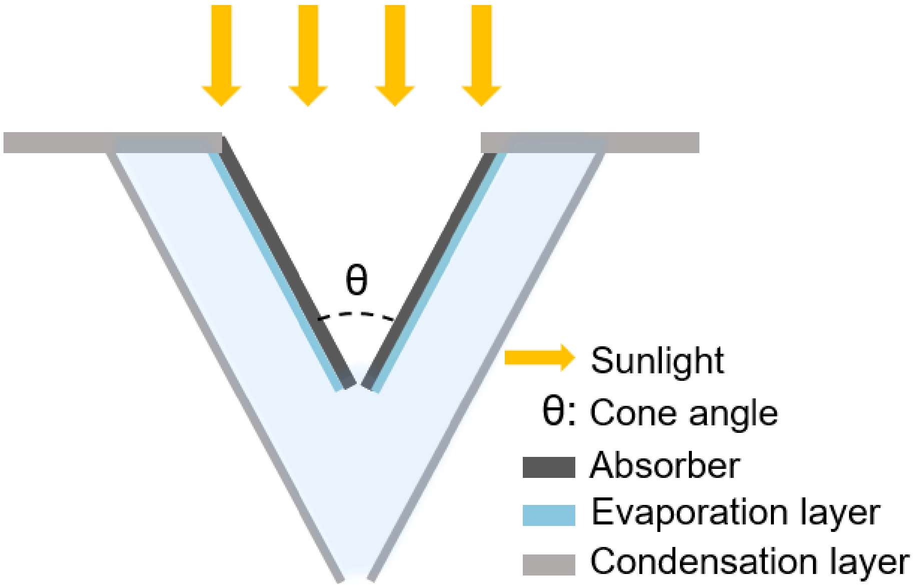

Supplementary Fig. 4 | Diagram illustrating the cone angle of the asymmetric tapered structure. The cone angle is fine as the angle between the central axis of the system and the tapered sides of the cone-like structure.

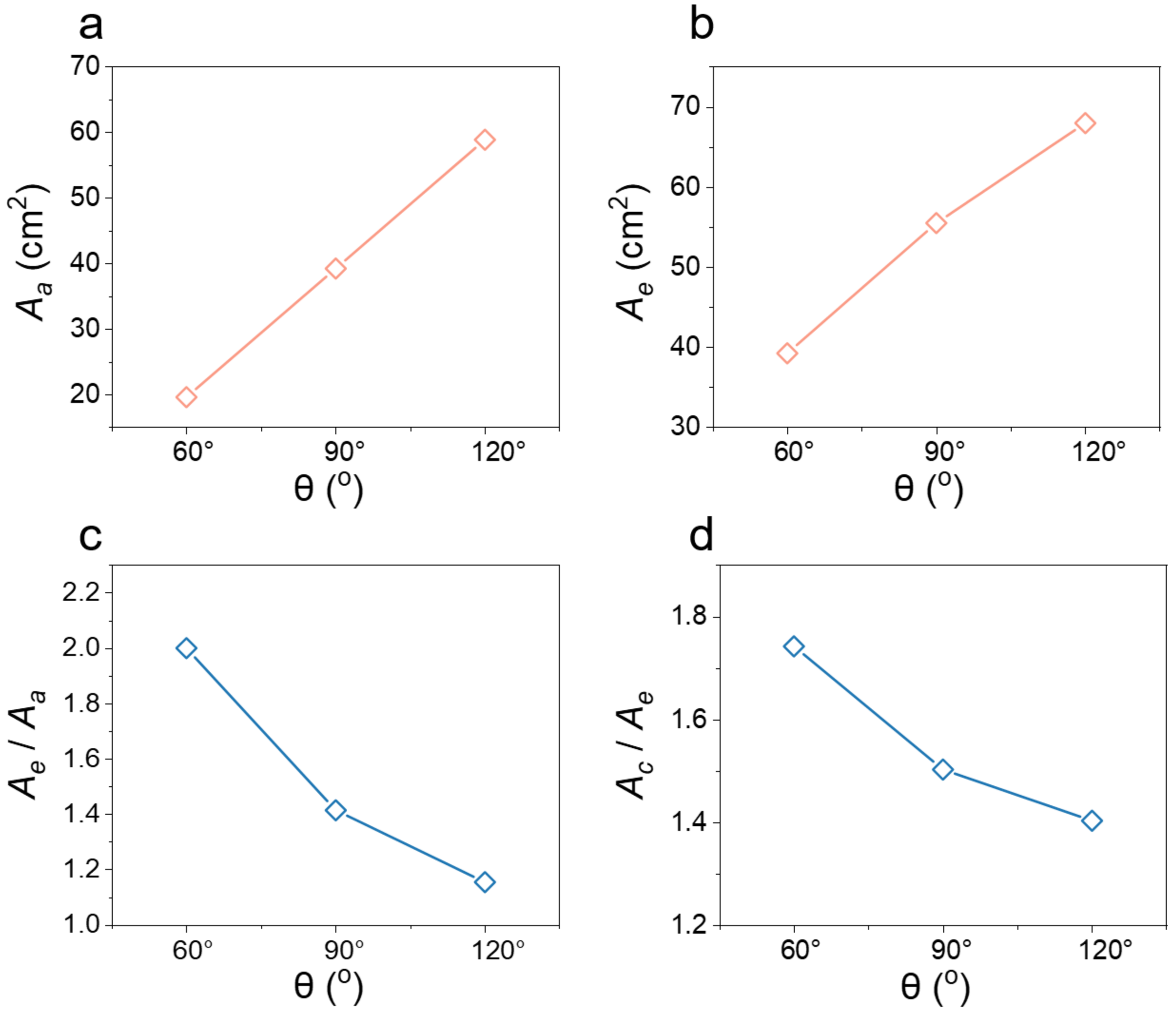

Supplementary Fig. 5 | The geometric parameters single-stage device with different conical apex angle. a, The light absorption area of single-stage device with conical apex angle of 60°, 90°, and 120°. **b,** The evaporation area of single-stage device with conical apex angle of 60°, 90°, and 120°. **c,** The ratio of evaporation and light absorption area. **d,** The ratio of condensation and evaporation area.

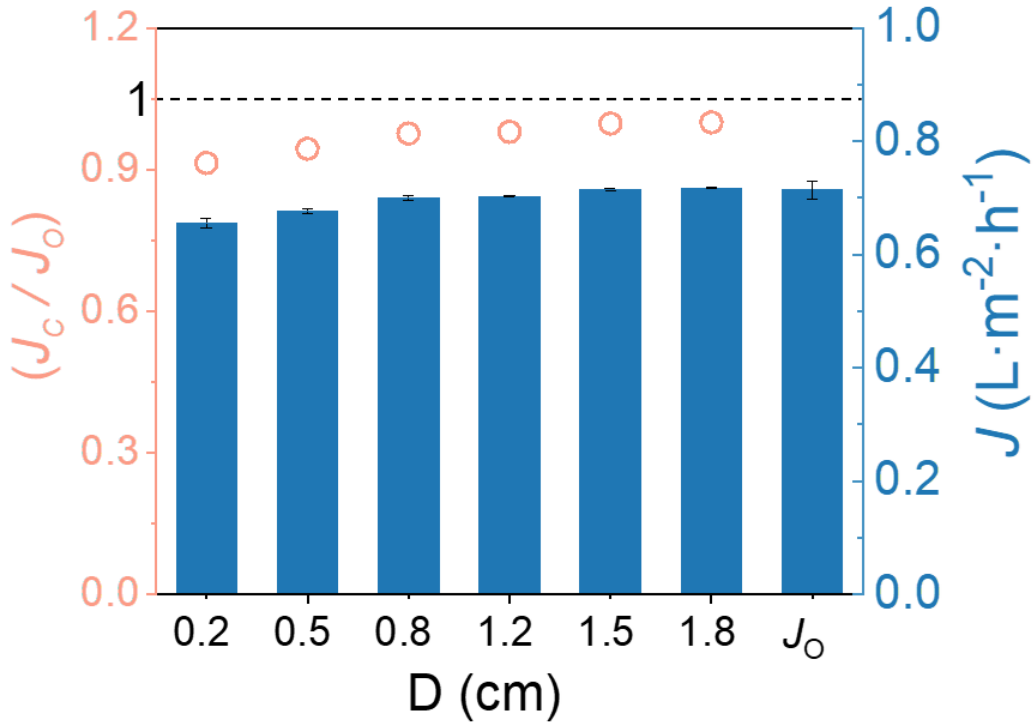

Supplementary Fig. 6 | The vapor production in open system (J_O) and closed chamber (J_C). J_C/J_O increases progressively with mass transfer gap, reaching 0.98 at the optimal mass transfer gap of 8 mm and stabilizing around 0.99. Error bars represent the s.d. (n = 3) and data are presented as mean values ± s.d

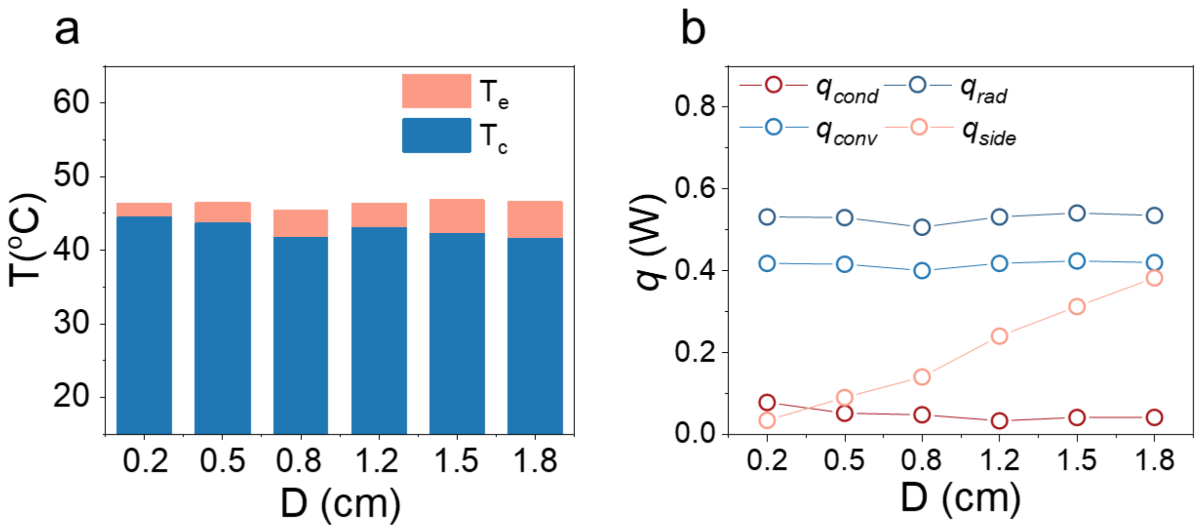

Supplementary Fig. 7 | The parasitic heat transfer of single-stage device with different mass transfer gap. a, The temperature of evaporation and condensation surface in single-stage device with different mass transfer gap. **b,** The conduction, convection, and radiation heat loss of single-stage device with different mass transfer gap.

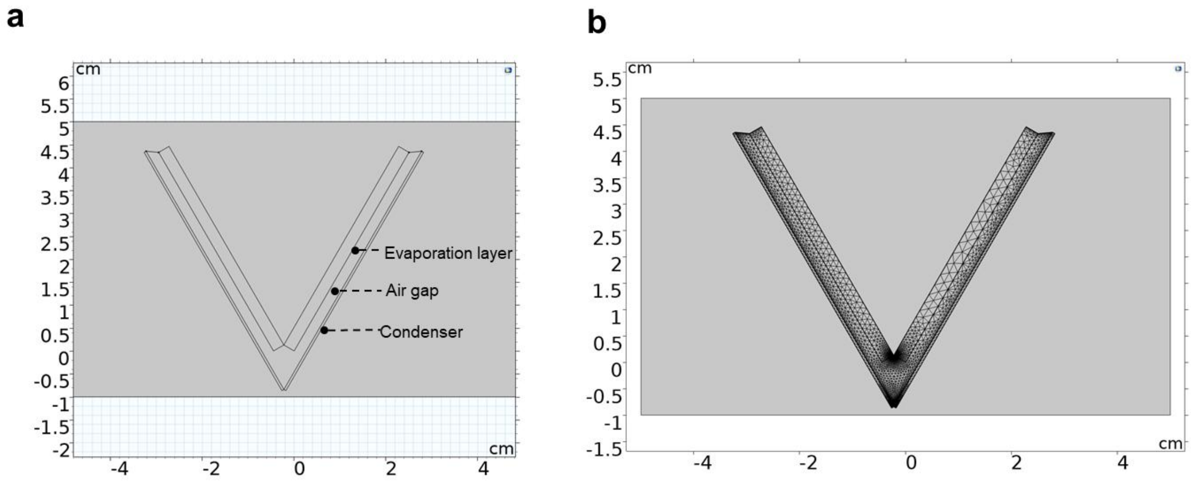

Supplementary Fig. 8 | The geometrical model of the asymmetric tapered multistage solar membrane distillation system. a, Two-dimensional geometrical model of the asymmetric tapered multistage solar membrane distillation system. **b,** Partial meshing of the asymmetric tapered multistage solar membrane distillation system in finite element simulation.

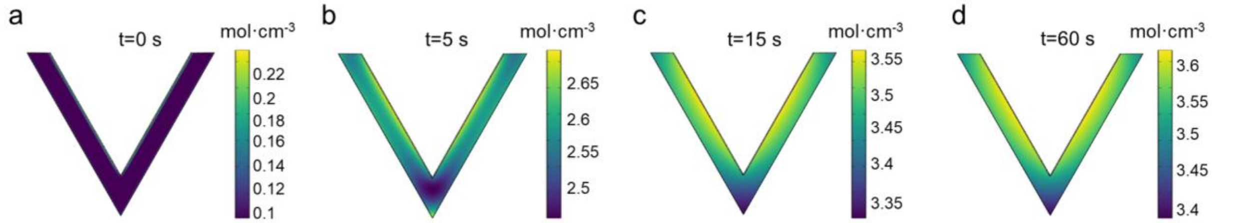

Supplementary Fig. 9 | Vapor distribution in the mass transfer chamber at different time intervals. a, at 0 s, **b,** at 5 s, **c,** at 15 s, and **d,** at 60 s.

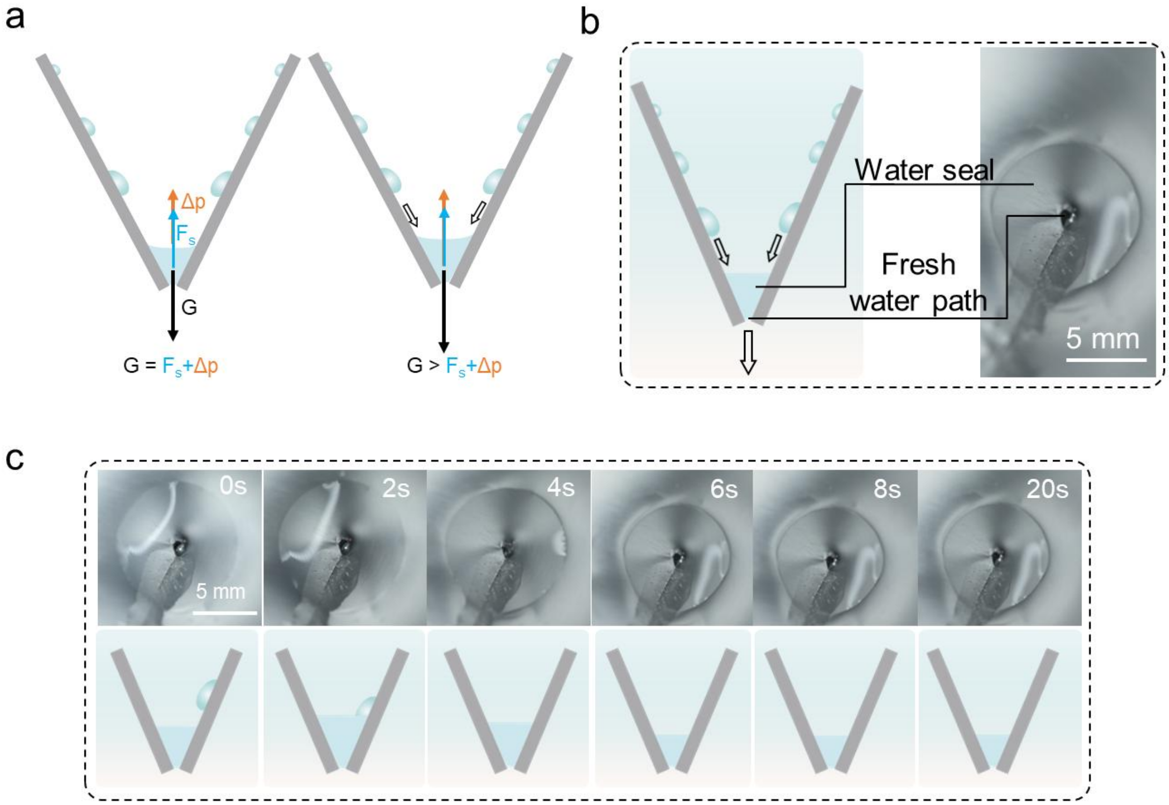

Supplementary Fig. 10 | The diagram and photograph of the mechanism of liquid seal. a, The mechanism of the liquid seal. **b,** The design and photograph of the liquid seal. Benefit from the surface tension, a stable liquid seal with a diameter of approximately 8 mm is formed on the millimeter-scale hole at the bottom of the cone. **c,** The photograph of the process during which the liquid seal remains stable even after water collection.

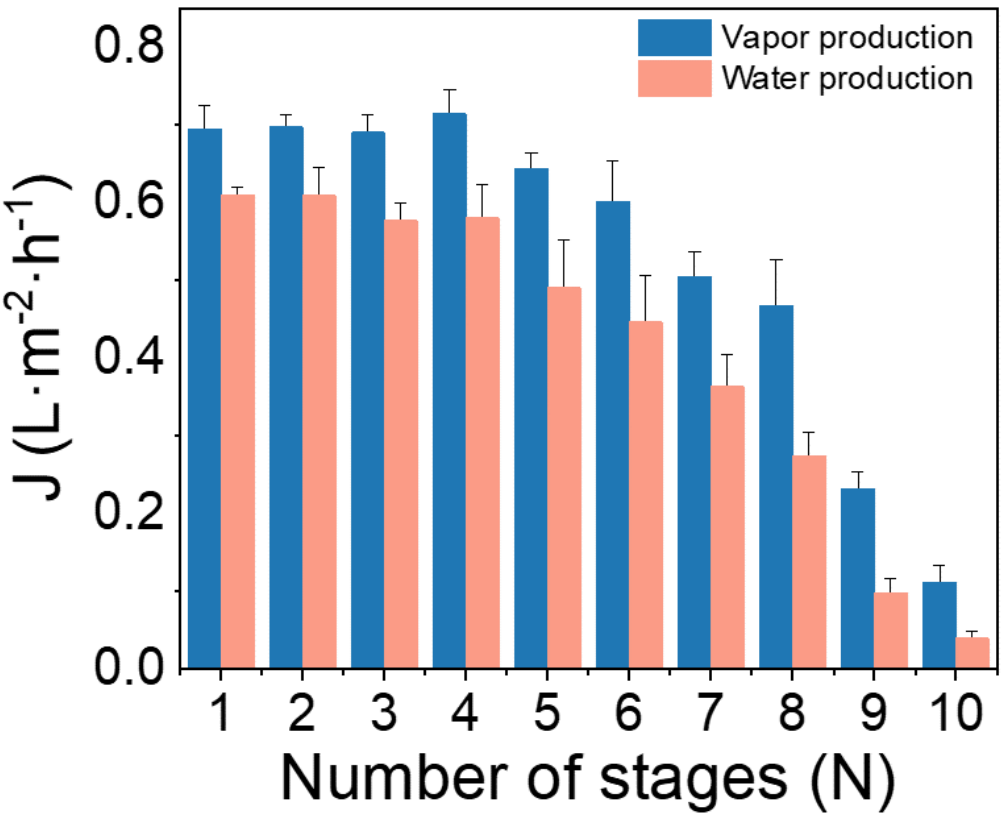

Supplementary Fig. 11 | The vapor and water production of ten-stage asymmetric tapered solar still with a uniformed D of 8 mm. Vapor production initially increases with the number of stages, and eventually decreased due to the diminishing evaporation temperature. The water production does not mirror the trend of vapor production and progressively decreases as the number of stages increases. Error bars represent the s.d. (n = 3) and data are presented as mean values ± s.d.

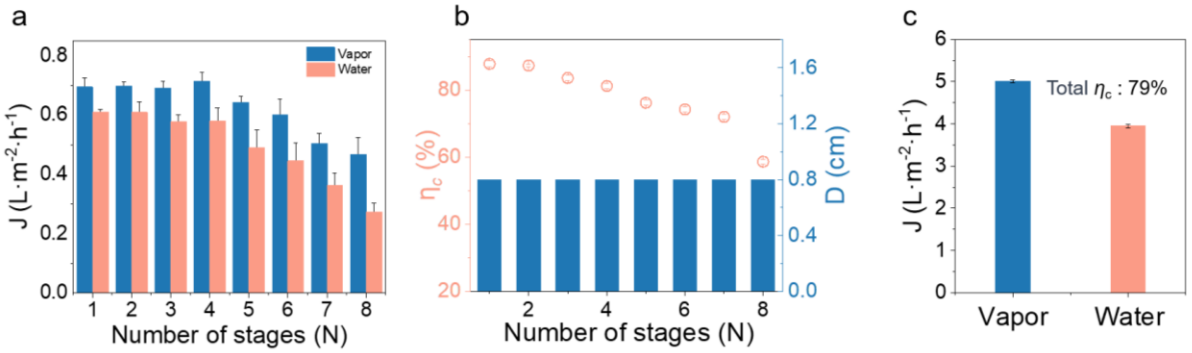

Supplementary Fig. 12 | The performance of optimized eight-stage asymmetric tapered solar still with a uniformed mass transfer gap of 8 mm. a, The vapor and water production of each stage. **b,** The vapor-to-water conversion efficiency of each stage. **c,** The total vapor and water production. Error bars in **a** represent the s.d. (n = 3) and data are presented as mean values ± s.d.

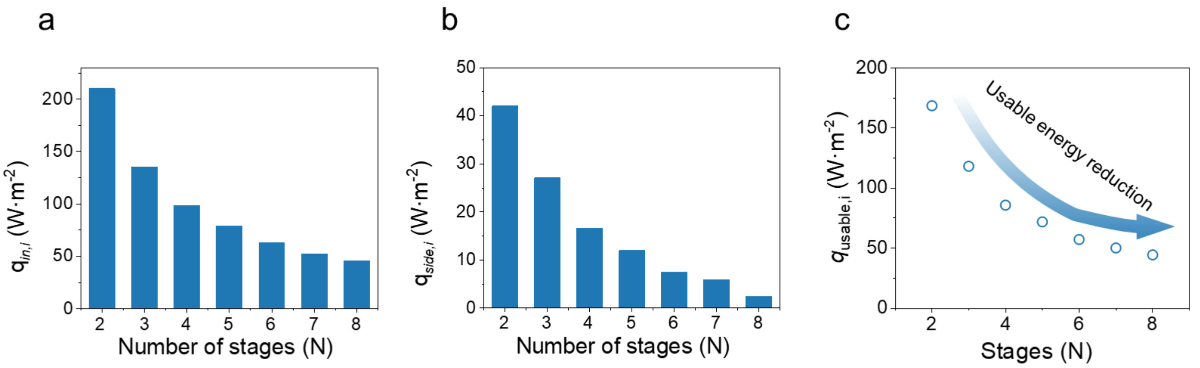

Supplementary Fig. 13 | The input energy and heat loss of each stages. a, The input energy of each stage. **b,** The heat loss of each stage. **c,** The heat utilized for evaporation gradually decreases with increasing stage number. The arrow indicates reducing usable energy trend.

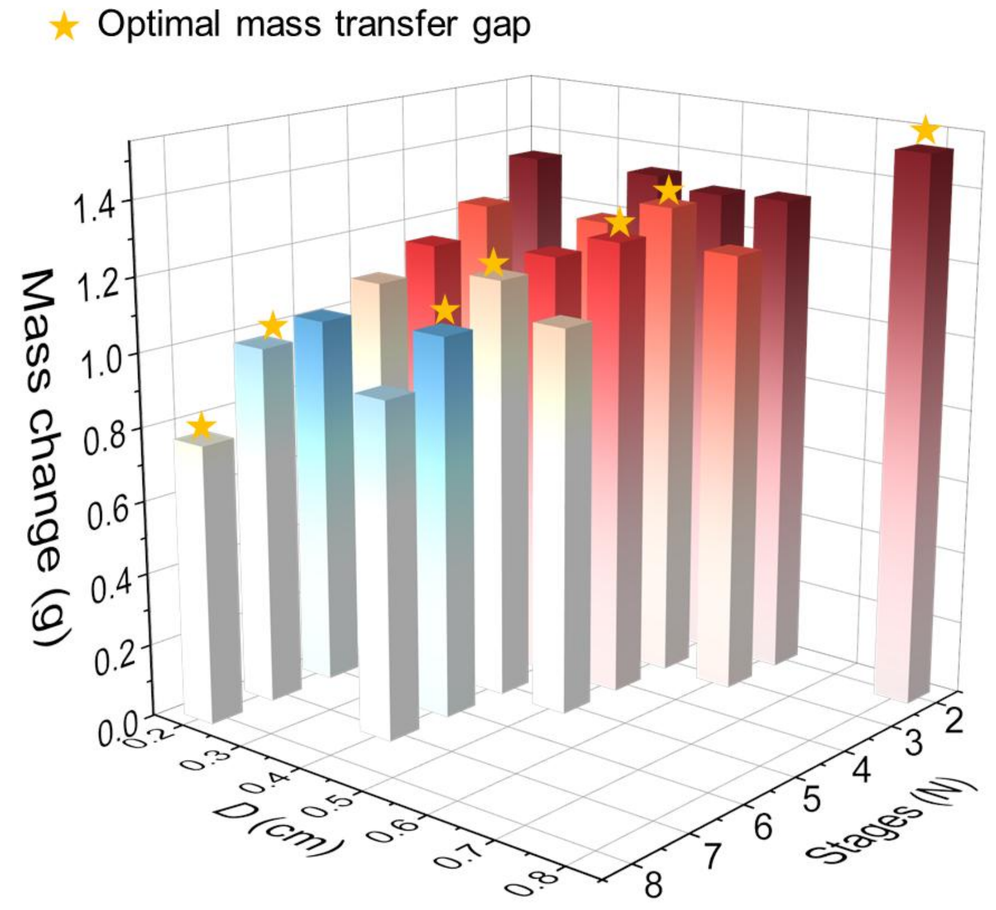

Supplementary Fig. 14 | The water production of each stage in eight-stage device with different mass transfer gap.

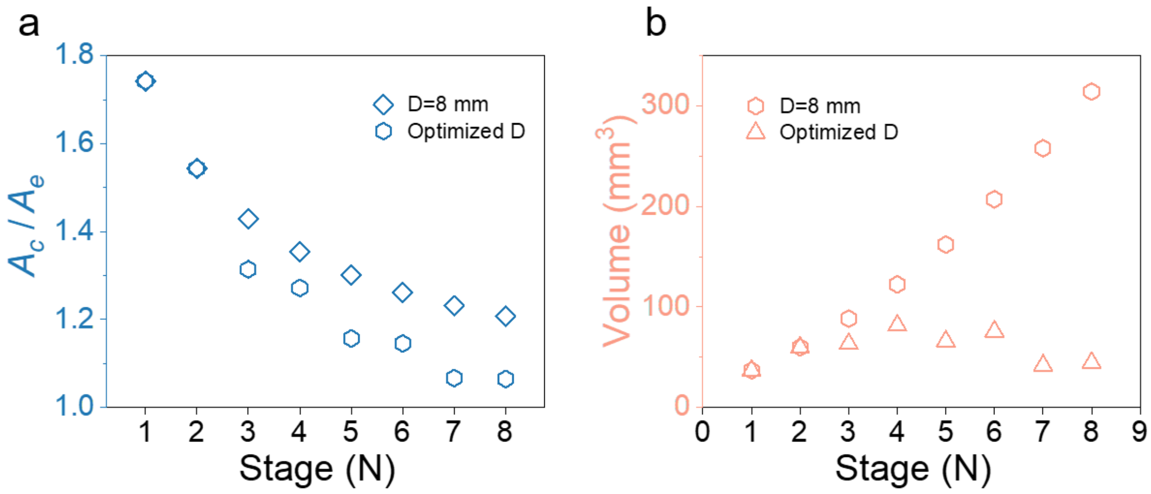

Supplementary Fig. 15 | The comparison of 8-stage asymmetric tapered solar still with a uniformed mass transfer gap of 8 mm and with a gradually decreasing mass transfer gap. a, The ratio of condensation area and evaporation area of each stage in these two devices. **b,** The Volumes of each stage in these two devices. The chamber volumes exhibit an initial slight increase followed by a decline, closely matching the vapor production trend across the stages.

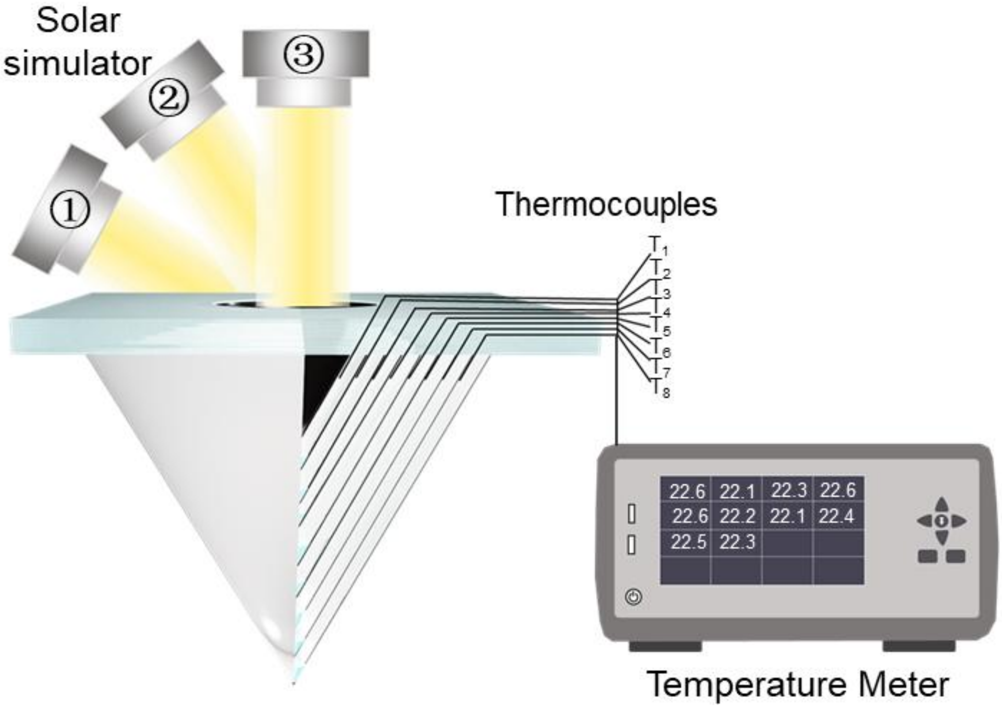

Supplementary Fig. 16 | The diagram of the setup under 1-sun illumination with different solar elevation angle. The sunlight is provided by a solar simulator, and the temperature is recorded by a temperature meter.

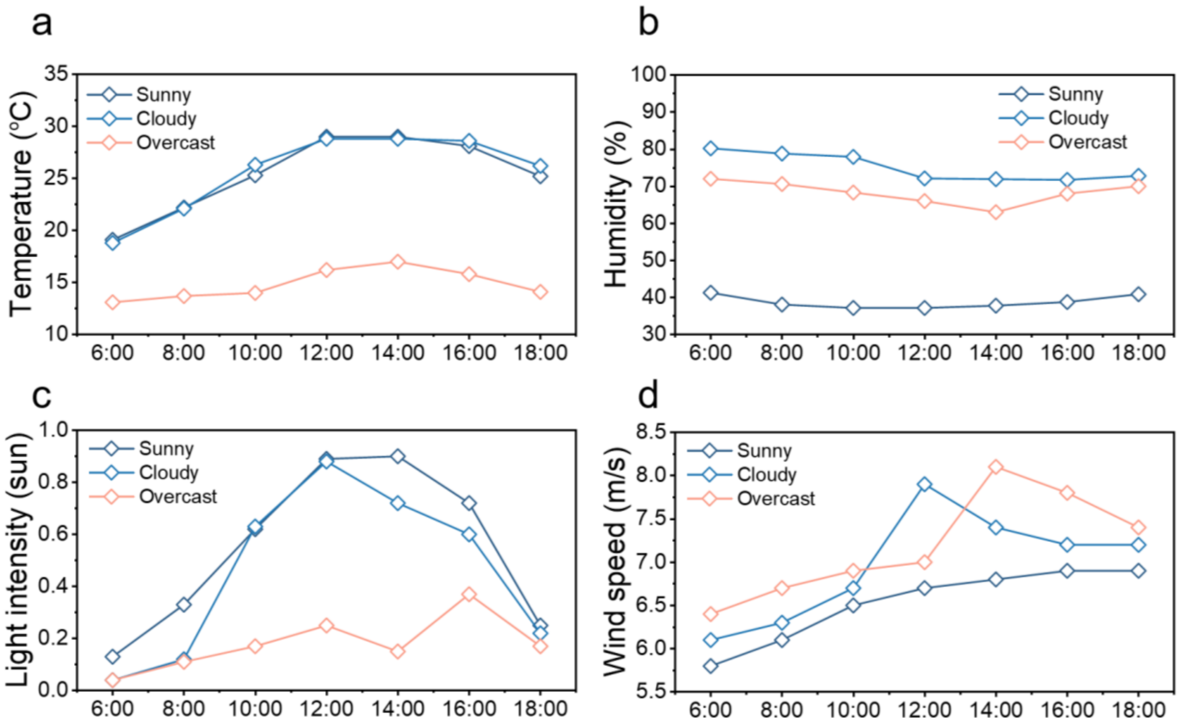

Supplementary Fig. 17 |The weather parameters of the three days with different climate conditions. a, The ambient temperature **a**, humidity **b**, light intensity **c** and wind speed **d** of the three days.

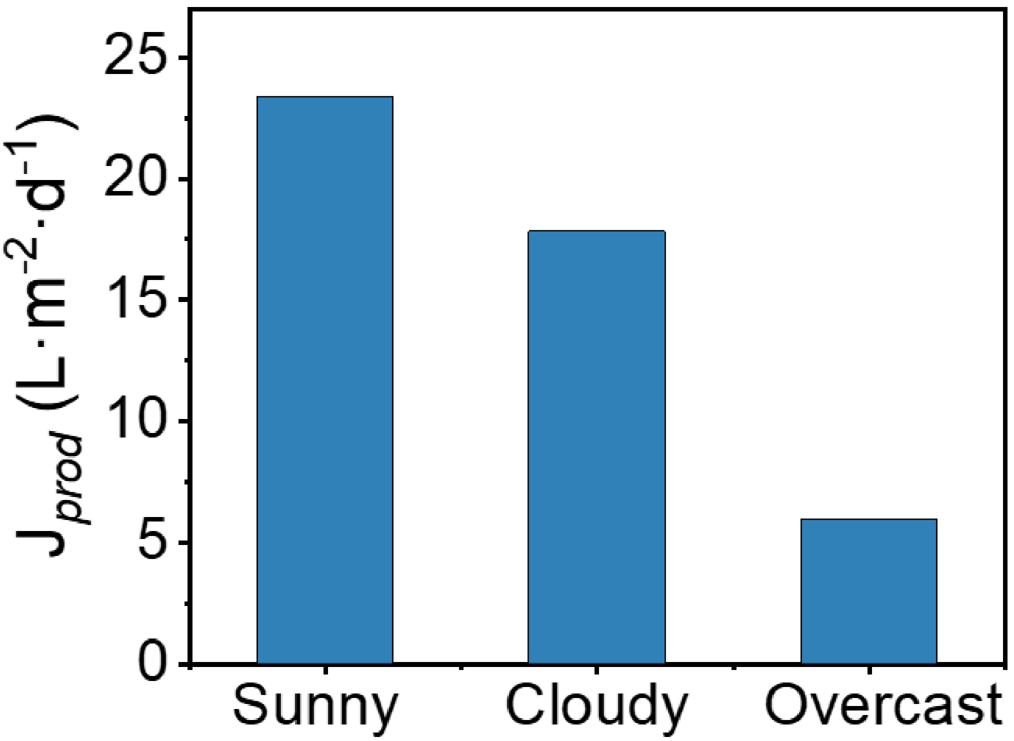

Supplementary Fig. 18 | The water production of the three days with different climate conditions.

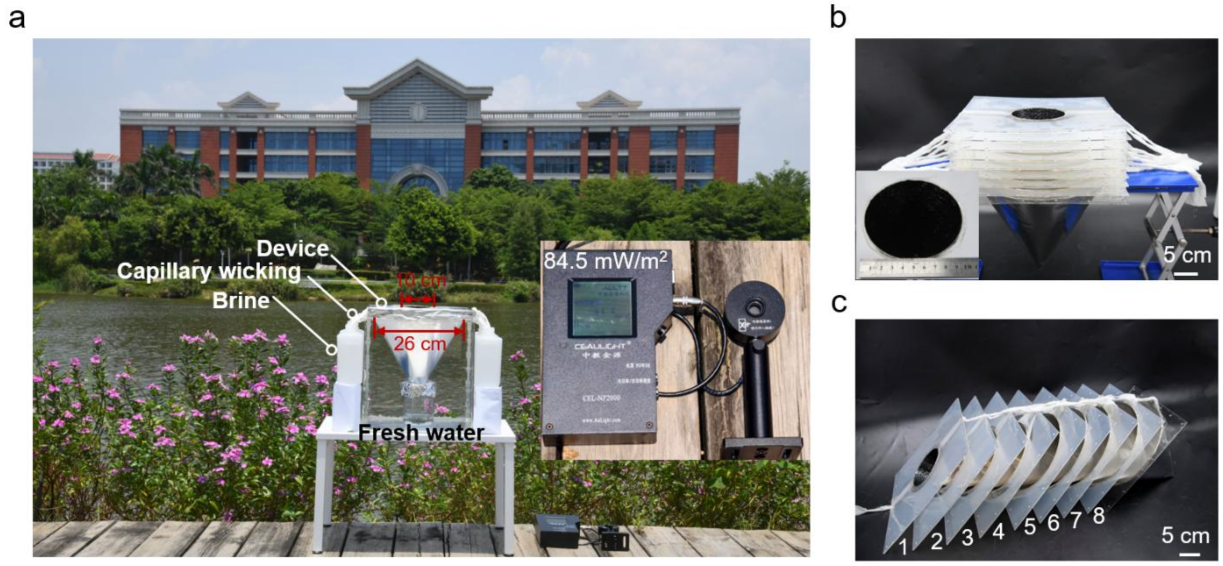

Supplementary Fig. 19 | The photograph of scaled-up eight-stage device. a, The photograph of outdoor test at the noon of sunny summer day. The illustration shows the light intensity. **b,** The front view of the disassembled structure of the scaled-up eight-stage device. **c,** Side view of the disassembled structure of the scaled-up eight-stage device.

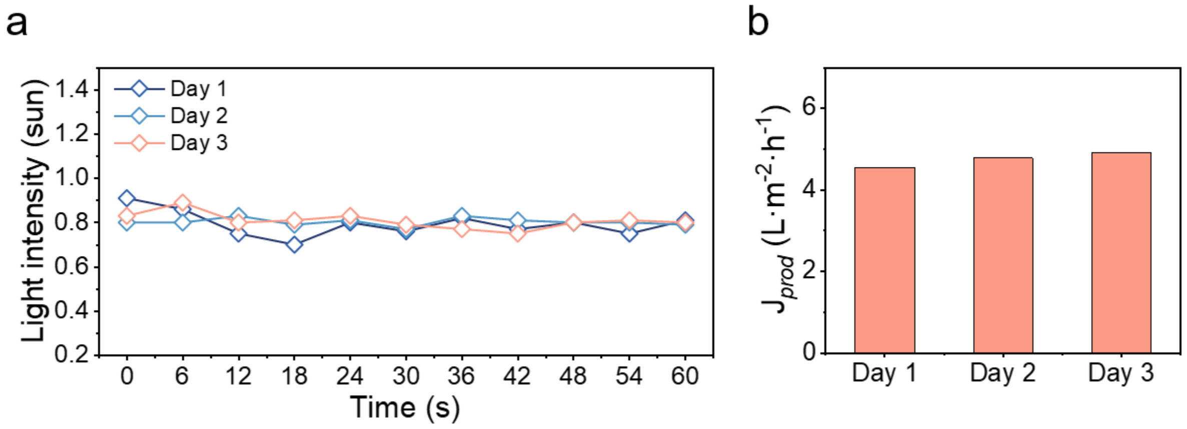

Supplementary Fig. 20 | The light intensity and water production at the noon of three sunny days. a, The light intensity of the three sunny days. **b,** The water production of the scaled-up device with twice the original size in the three days.

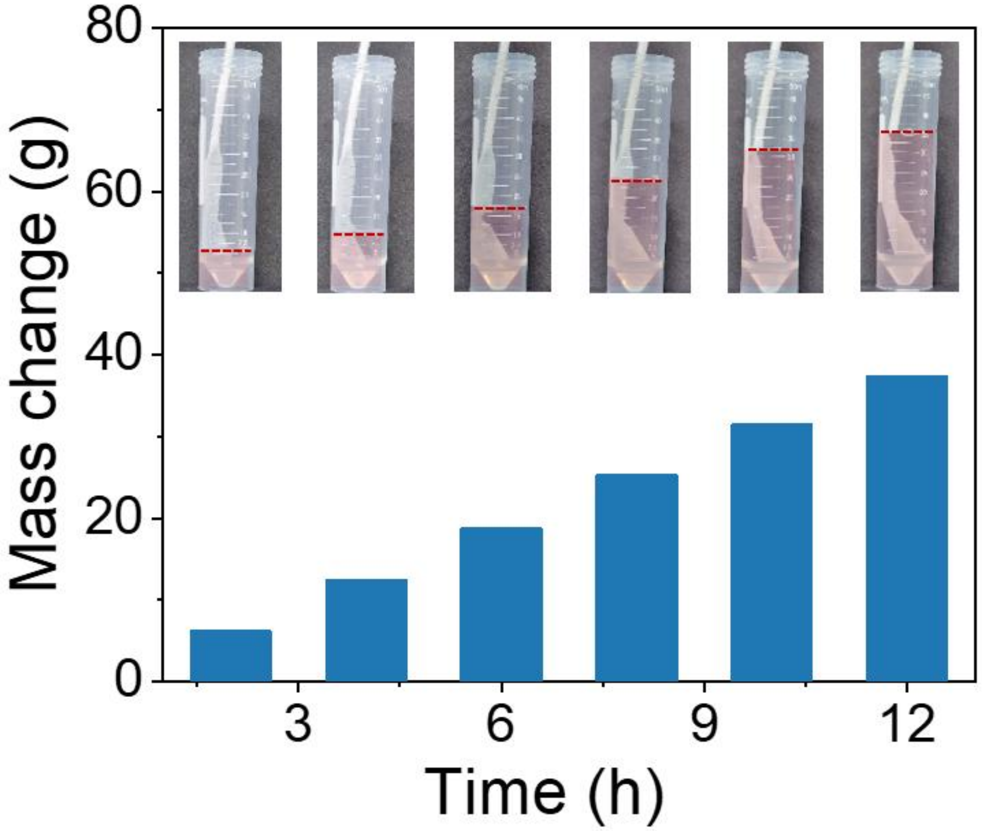

Supplementary Fig. 21 | The collected brine in the low-level side during the dark. The volume of collected brine gradually increases over time as the system continues to operate. The insets are the photographs of the collected water at different time.

Supplementary Tables

Supplementary Table 1. The parameters of single-stage device with different D.

D (cm)	T_e (℃)	T_c (℃)	T_a (℃)
0.2	46.4	44.5	25.1
0.5	46.5	43.7	25.1
0.8	45.4	41.7	25.1
1.2	46.4	43.1	25.1
1.5	46.9	42.3	25.1
1.8	46.6	41.6	25.1

Supplementary Table 2. The parameters of multistage device.

Stages (N)	T_e (℃)	T_c (℃)	T_a (℃)	t (mm)
1	45	42.5	25.1	4
2	41.9	39.8	25.1	4
3	38.3	36.8	25.1	4
4	36.2	34.9	25.1	4
5	34.5	33.2	25.1	4
6	32.8	31.6	25.1	4
7	31.1	29.9	25.1	4
8	29.3	28.4	25.1	4

Supplementary Table 3. The data of state-of-the-art multistage solar membrane distillation devices used for comparison

Reference	Number of stages	Water production ($L \cdot m^{-2} \cdot h^{-1}$)	Feedwater type	Heat Flux ($kW \cdot m^{-2}$)
Ref.20	3	2.23	Seawater	1
Ref.28	4	1.98	3.5 wt% NaCl	1
Ref.26	5	2.45	Seawater	1
Ref.38	6	2.2	Seawater	1
Ref.37	7	3.22	Seawater	1
Ref.39	8	2.25	3.5 wt% NaCl	1
Ref.25	8	2.63	Seawater	1
Ref.29	8	3.61	Seawater	1
This work	8	4.32	Seawater	1
Ref.27	10	3.82	3.5 wt% NaCl	1
Ref.25	10	3.17	Seawater	1
Ref.36	10	4.03	3.5 wt% NaCl	1

Supplementary Reference

1. Zhang, L, et al. Modeling and Performance Analysis of High-Efficiency Thermally-Localized Multistage Solar Stills. *Appl. Energy*. **266**, 114864 (2020).
